# Dementia and the history of disease in older adults in community

**DOI:** 10.1186/s12889-023-16494-x

**Published:** 2023-08-16

**Authors:** Yuan Wang, Honglian Xu, Zihan Geng, Guiling Geng, Feng Zhang

**Affiliations:** 1 Medical College of Nantong University, 19 QiXiu Road, Nantong City, Jiangsu Province China; 2 Nantong North Rehabilitation Hospital, Nantong City, Jiangsu Province China

**Keywords:** Dementia, Medical history, Older adults, Community

## Abstract

**Introduction:**

Many studies have revealed the effect of medical history on dementia. The aim of this study was to explore the relationship between the history of disease and onset of dementia.

**Methods:**

This was a multi-center, cross-sectional study, with 2595 older adults enrolled. The onset of dementia was evaluated with Revised Hasegawa Dementia Scale (HDS-R). The diagnosed diseases after the age of 40 of the participants were investigated, including respiratory system diseases, digestive system diseases, cardiovascular diseases, endocrine disorders, genitourinary system diseases, nervous system disease, sensory system diseases, dental/oral diseases, bone/joint diseases and mental illnesses.

**Results:**

Data of 2458 older adults were analyzed. Univariate analysis showed that diabetes, thyroid disease, mental illness, hearing loss, stroke, dental/oral disease, Denture use, fracture/osteoporosis, kidney disease and number of diseases were risk factors for dementia. After controlling for demographic sociological variables, diabetes, dental/oral disease, and denture use were independent risk factors for dementia. Thyroid disease (*P* = 0.313), mental illnesses (*P* = 0.067), hearing loss (*P* = 0.595), stroke (*P* = 0.538), fractures/osteoporosis (*P* = 0.069), kidney disease (*P* = 0.168) were no longer significant to dementia.

**Conclusion:**

Diabetes, dental/oral disease and denture use were main risk factors for dementia.

## Introduction

With the global aging trend accelerating, dementia has been identified as the greatest global challenge for public health and social care [1]. The number of people with dementia around the world is expected to increase to 66 million by 2030 [2]. The proportion of people with dementia living in low and middle income countries (LMICs) is likely to reach 71% by 2050 [3]. China has the largest population with dementia [4], accounting for 20% of global dementia patients [5]. Globally, the cost for dementia is over US $800 billion annually [6], which makes the health care systems be in danger of becoming overwhelmed [7].

The mechanism of dementia remains unclear. It is related to aging and degeneration of the nervous system, featuring amyloid plaques, neurofibrillary tangles and neuronal loss in the temporal lobe and neocortex of the brain [8]. Known risk factors for dementia included heredity, aging, gender, social isolation [9], socioeconomic status [10], abdominal obesity [11], physical inactivity [11], excessive intake of saturated fat [12], cardiovascular diseases [13], cerebrovascular diseases [13], dyslipidemia, diabetes [14], brain trauma [15] and depression [16].

Heredity, aging and gender are main immutable factors for dementia. Therefore, increasing studies have attached importance to the prevention and treatment of these risk factors to prevent dementia [17]. Wei et al. reported that stroke, lack of fruit, household financial management, a history of cardiovascular disease, hypertension, and physical exercise were also associated with dementia [18]. With population attributable fractions (PAF) method, Mukadam et al. estimated that about 35% of dementia cases could be prevented by targeting at nine modifiable risk factors, including early life education, midlife hypertension, obesity, hearing loss, old-age smoking, depression, physical inactivity, diabetes, and social isolation [19].

Many studies have revealed the impact of medical history on dementia [20]. If one particular disease leading to dementia was eliminated, such as hearing loss, hypertension, depression or diabetes, the incidence of dementia was reduced by 9.1%, 2.0%, 4.0%, and 1.2% respectively [19]. Warren et al. argued that more than 20 years before the beginning of dementia, the preclinical symptom of dementia has occurred [21]. Individuals with poor pulmonary function were at an increased risk of dementia [22]. Proton Pump Inhibitors may account for the relationship between digestive system diseases and dementia [23]. VEGF ligand-receptor interactions and anti-inflammatory cytokine pathways were related to dementia among older adults with cardiovascular diseases [24]. Dementia could also be caused by altered hormone levels in endocrine disorder [25]. As to nervous system disease, the tight coupling between neuronal activity and cerebral blood flow is lost in dementia, which may play a key role in cognitive dysfunction [26]. Sensory impairment could also be associated with factors related to resilience against dementia [27]. When it comes to dental/oral diseases, previous literature showed that the number of lost teeth, lack of oral hygiene, bacterial dental plaque accumulation, gingival inflammation and mastication were related to the incidence of dementia [28–30]. Neuroinflammation activated the microglia and proinflammatory cytokines, and then triggered irreversible neurodegenerative deterioration [31]. Concerning mental illnesses, chronic stress and inflammation combine to compromise vascular and brain function. Increasing proinflammatory cytokines and microglial activation drive brain pathology, which may progress to dementia [32]. Therefore, tackling the complicated diseases in midlife years before the development of dementia is appropriate and essential. There are many tools for screening dementia, such as Montreal Cognitive Assessment [33], Modified Mini-Mental State Examination [34] and the Revised Hasegawa Dementia Scale (HDS-R). HDS-R, As a simple dementia screening scale and an ideal tool for screening dementia, HDS-R is simple, not affected by the educational level of the respondents [35, 36]. However, few studies focus on the relationship between dementia and medical history systemically.

In this study, a cross-sectional survey was conducted to investigate the medical history after the age of 40 and the status of dementia in older adults in community to explore the correlation between dementia and medical history.

## Methods

### Study design

This was a multi-center, cross-sectional research.

### Setting and sample

A two-stage sampling method was adopted. During the first stage, communities stratified by regions were randomly selected in Nantong, an eastern city in China. According to data from Nantong Civil Affairs Bureau in 2018, Nantong City was divided into three districts. There were 21 communities in all. The number of populations in all communities was 1 219 700. Participants over 60 years old accounted for 19.73% (240,600). One community was randomly selected from each district according to a computer-generated random number.

Second, all people over 60 years old in the selected community were numbered, and 1000 people were selected randomly. Then, a telephone appointment was made to evaluate their inclusion criteria and confirm the permission of a home visit.

### Participants

We recruited people over 60, who were able to listen and speak. If a family has more than one older adults, only one of them was investigated. The exclusion criteria were: 1) cognitive dysfunction; 2) blindness; 3) aphasia; 4) deafness; or 5) people who have been diagnosed with dementia. A meeting to calibrate was held prior to the survey. The survey was conducted by five full-time investigators from February to June in 2019.

### Ethical consideration

The protocol was approved by the Ethics Committee of the Affiliated Hospital of Nantong University (2018-K042) and registered in Clinical Trial Registry (ChiCTR1900020923). All methods were carried out in accordance with relevant guidelines and regulations. Verbal and written information regarding the aims, procedures, potential risks, and benefits were introduced to all participants. They were informed of the freedom to quit unconditionally from our research. Written informed consent was obtained from all subjects.

### Measurements

#### Dementia

The Revised Hasegawa Dementia Scale (HDS-R) was applied to older adults as a Dementia Screening Instrument. It is widely used all over the world as a common diagnostic test for dementia [37]. This scale consists of 9 simple questions with a total score of 30 points, including age, time orientation, location orientation, instant recall, continuous reduction, recall digits backward, recalling three words, retelling five objects, and stating the names of vegetables [38]. A score of 20/21 on the HDS-R is a determinate index for discriminating between normal cognition and dementia. The sensitivity is 0.90, and the specificity is 0.82. People who scored less than 21 were defined as HDS-R positive.

#### Medical history

We investigated the older adults or their family members on the diagnosed diseases after the age of 40, including respiratory system diseases, digestive system diseases, cardiovascular diseases, endocrine disorders, genitourinary system diseases, nervous system disease, sensory system diseases, dental/oral diseases, bone/joint diseases and mental illnesses.

Respiratory system diseases mainly included asthma and COPD (chronic obstructive pulmonary disease). Digestive system diseases included chronic gastritis, peptic ulcer, esophagitis, enteritis, cirrhosis, pancreatitis, and cholecystitis. Cardiovascular diseases mainly included hypertension, angina pectoris and myocardial infarction. Endocrine/metabolic disorders included 1) diabetes; 2) hyperlipidemia, included hypertriglyceridemia and hypercholesterolemia; 3) Thyroid disease included hyperthyroidism and hypothyroidism. Genitourinary system diseases included 1) kidney disease, including chronic urinary tract infections, chronic nephritis and uremia; 2) Prostate disease, refers to benign prostatic hyperplasia; and 3) Gynecological diseases, included chronic cervicitis, vaginitis, pelvic inflammatory endometriosis, uterine fibroids, and dysfunctional uterine bleeding. Nervous system disease included 1) stroke, included cerebral hemorrhage and cerebral infarction; and 2) Parkinson's disease. Sensory system diseases included 1) eye diseases included glaucoma, cataracts, and retinopathy; and 2) Ear disease refers to hearing loss. Dental/oral diseases included denture use, periodontitis, periapical periodontitis, residual roots, and tooth loss (excluded denture installation). Bone and joint diseases included 1) fractures and osteoporosis; and 2) joint diseases, included rheumatoid joints and arthritis. Mental illnesses included depression and schizophrenia. Other diseases refer to 1) anemia; 2) malignant tumor; and 3) trauma of the other parts.

### Data collection

The demographic information such as gender, age, education, career, and medical history was initially obtained from the registration data in the Community Health Service Center. The HDS-R score was obtained through a face-to-face evaluation of the older adults by our professional investigators. Their family members, housework allocation, and social activity, smoking and drinking were attained by self-report. The data of medical history and career experience were acquired both from the older adults themselves and their family members (usually their children or partner) in consideration of the recall bias of the older adults. All information of the older adults who lived alone needed to be checked with their children or relatives through telephone interview.

### Data analysis

Two persons were engaged in data entry separately. The input errors were checked by Epidata 3.1. The statistical analysis was performed with SPSS 25.0. Missing data were dealt with case-wise deletion method given that the proportion of missing data was small (0 ~ 5.28%). Chi-square test was used for grouped data, and the rank-sum test was adopted for ranked data. Multivariate regression was adopted for the analysis of the relationship between dementia and medical history. In Model 1, we controlled age, education, house work, social activity, spouse, smoking and drinking. Because age is a key variable for dementia, a subgroup analysis was conducted based on age groups (Age: 60–70, 71–80, and > 81) after controlling for education, house work, social activity, spouse, smoking and drinking. Because of the small sample size in population more than 90 years old, we combined the age group of 81–90 and > 90.

## Results

**Fig. 1:**
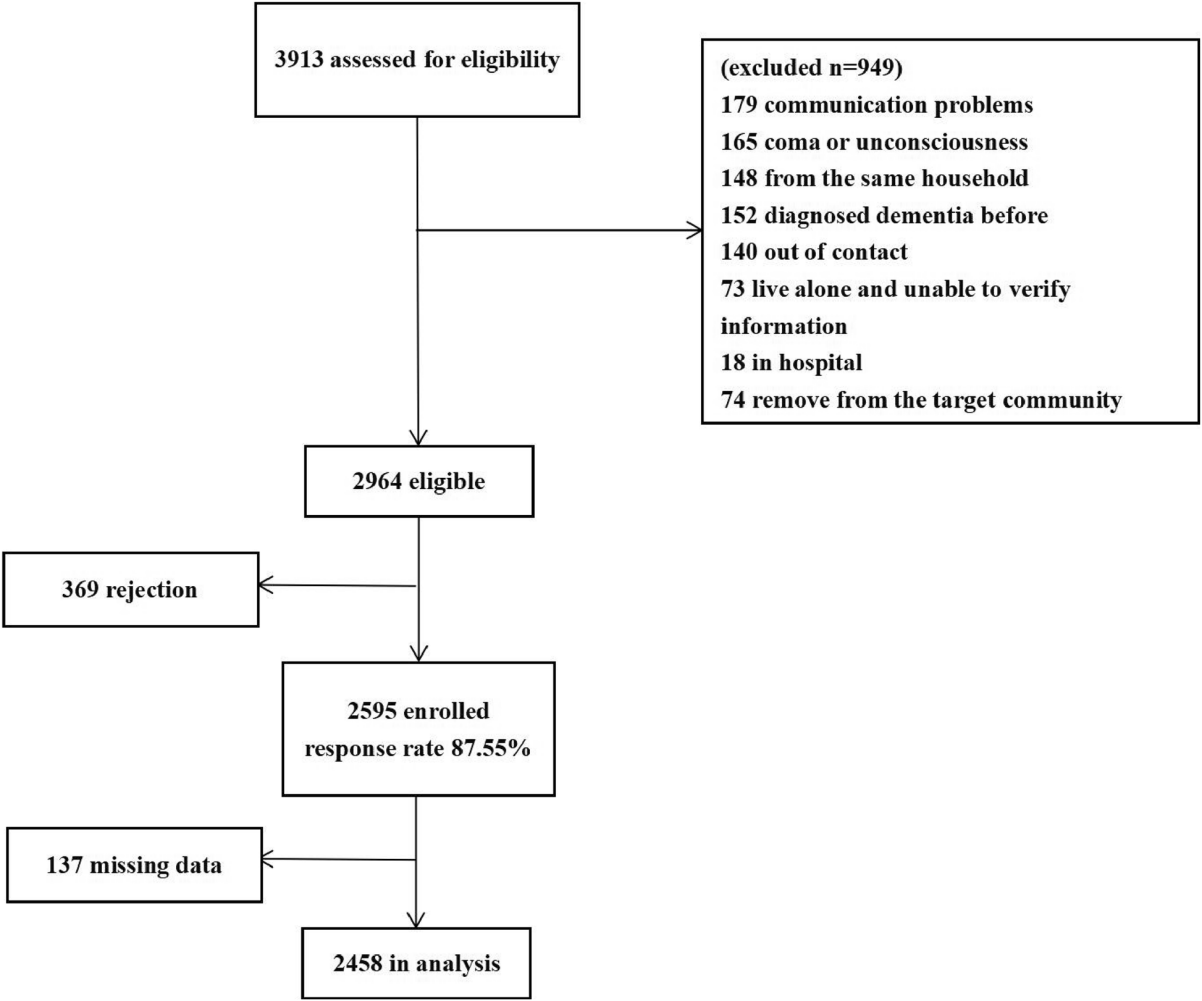
Flow chart of participants from eligibility to data analysis

Of the 3913 evaluated older adults, 2964 were eligible. 369 people (12.45%) refused to take part in this study (Fig. 1). No difference was found regarding baseline information between the recipients and those who declined: gender ($\chi^{2}$ = 0.42 *p* = 0.516), education ($\chi^{2}$ = 1.17 *p* = 0.557), and employment ($\chi^{2}$ = 6.14 *p* = 0.189). However, participants who declined were younger (75.56 ± 7.89 year VS. 67.28 ± 5.64 year, *t* = -19.44 *p* < 0.01).

Among the 2595 enrolled, 137 cases (5.28%) have missing data. Therefore, data of 2458 participates were analyzed. Among them, 559 (22.7%) older adults were evaluated as potential dementia by HDS-R.

Based on the screening of HDS-R, age was the predominant risk factor for dementia. The prevalence of dementia in the older adults were 14.67% (119/811), 18.81% (186/989), 34.09% (196/575) and 69.88% (58/83) in population aged 60–70, 71–80, 81–90, and > 90. Besides, long-time separation from spouse could also lead to dementia. High level of education, post-retirement work, housework allocation, and more types of housework, social activity, living with spouses, smoking, and drinking were protective factors of dementia (Table 1).

**Table 1:** Demographic information for the older adults (*n* = 2458)

	Full sample (*n* = 2458)	Dementia (*n* = 559)	None (*n* = 1899)	*P*
Gender				0.591
Male	1110(45.16)	258(46.15)	852(44.87)	
Female	1348(54.84)	301(53.85)	1047(55.13)	
Age, years				< 0.001
60 ~ 70	811(32.99)	119(21.29)	692(36.44)	
71 ~ 80	989(40.24)	186(33.27)	803(42.29)	
81 ~ 90	575(23.39)	196(35.06)	379(19.96)	
> 90	83(3.38)	58(10.38)	25(1.31)	
Education				0.001
Illiteracy	444(18.06)	129(23.08)	315(16.59)	
Primary school	1246(50.69)	278(49.73)	968(50.97)	
Middle school and above	768(31.25)	152(27.19)	616(32.44)	
Career				
Public institution	447(18.19)	91(16.28)	356(18.75)	0.589
Enterprise	317(12.90)	79(14.13)	238(12.53)	
Worker	311(12.65)	73(13.06)	238(12.53)	
Farmer	1275(51.87)	294(52.59)	981(51.66)	
None	108(4.39)	22(3.94)	86(4.53)	
Number of career experience				0.342
0	108(4.39)	22(3.94)	86(4.53)	
1	2022(82.26)	458(81.93)	1564(82.36)	
2	264(10.74)	55(9.84)	209(11.01)	
> 2	64(2.60)	24(4.29)	40(2.11)	
Post-retirement work				< 0.001
Yes	417(16.97)	61(10.91)	356(18.75)	
No	2041(83.03)	498(89.09)	1543(81.25)	
Housework allocation				< 0.001
Yes	1147(46.67)	180(32.20)	967(50.92)	
No	1311(53.33)	379(67.80)	932(49.08)	
Type of housework				
0	1311(53.33)	379(67.80)	932(49.08)	< 0.001
1 ~ 2	887(36.09)	151(27.01)	736(38.76)	
> 2	260(10.58)	29(5.19)	231(12.16)	
Social activity				< 0.001
Yes	525(21.36)	85(15.21)	440(23.17)	
No	1933(78.64)	474(84.79)	1459(76.83)	
Living Partner				0.102
Live Alone	304(12.45)	80(14.31)	224(11.80)	
Live with couple only	905(36.82)	187(33.45)	718(37.81)	
Live with younger generation	1247(50.73)	292(52.24)	955(50.29)	
Total family Members, No.				0.074
1	72(2.93)	15(2.68)	57(3.00)	
2 ~ 5	1592(64.77)	346(61.90)	1246(65.61)	
> 5	794(32.30)	198(35.42)	596(31.38)	
Living with spouse				< 0.001
Yes	1861(75.71)	380(67.98)	1481(77.99)	
No	597(24.29)	179(32.02)	418(22.01)	
Separation time from spouse, years				0.000
< 1	1865(75.87)	386(69.05)	1479(77.88)	
1 ~ 3	153(6.22)	45(8.05)	45(2.37)	
4 ~ 5	92(3.74)	27(4.83)	27(1.42)	
> 5	348(14.16)	101(18.07)	237(12.48)	
Smoking				0.033
Yes	335(13.63)	61(10.91)	274(14.43)	
No	2123(86.37)	498(89.09)	1625(85.57)	
Drinking				0.008
Yes	507(20.63)	93(16.64)	414(21.80)	
No	1951(79.37)	466(83.36)	1485(78.20)	

Among all the diseases investigated, univariate analysis showed that diabetes, thyroid disease, mental illness, hearing loss, stroke, dental/oral disease, denture use, fracture/osteoporosis, and kidney disease were risk factors for dementia. More types of diseases were associated with an increased incidence of dementia. The prevalence of dementia in the older adults with one to two diseases, three to five diseases and more than 5 diseases were 20.47% (287/1402), 27.07% (160/591) and 41.75% (43/103) respectively (Table 2).

**Table 2:** Medical diseases and dementia (*n* = 2458)

	Total (*n* = 2458)	Dementia (*n* = 559)	None (*n* = 1899)	*P*
Diabetes				0.003
No	2005(81.57)	432(77.28)	1573(82.83)	
Yes	453(18.43)	127(22.72)	326(17.17)	
Hyperlipidemia				0.110
No	2179(88.65)	485(86.76)	1694(89.20)	
Yes	279(11.35)	74(13.24)	205(10.80)	
Thyroid disease				0.025
No	2431(98.90)	548(98.03)	1993(99.16)	
Yes	27(1.10)	11(1.97)	16(0.84)	
Mental illnesses				0.039
No	2433(98.98)	549(98.21)	1884(99.21)	
Yes	25(1.02)	10(1.79)	15(0.79)	
Parkinson's disease				0.114
No	2426(98.70)	548(98.03)	1878(98.89)	
Yes	32(1.30)	11(1.97)	21(1.11)	
Eye diseases				0.185
No	2217(90.20)	496(88.73)	1721(90.63)	
Yes	241(9.80)	63(11.27)	178(9.37)	
Hearing loss				0.000
No	2319(94.34)	509(91.06)	1810(95.31)	
Yes	139(5.65)	50(8.94)	89(4.69)	
Cardiovascular diseases				0.646
No	1195(48.62)	267(47.76)	928(48.87)	
Yes	1263(51.38)	292(52.24)	971(51.13)	
Stroke				0.001
No	2256(91.78)	494(88.01)	1762(92.79)	
Yes	202(8.22)	65(11.63)	137(7.21)	
Respiratory system diseases				0.398
No	2301(93.61)	519(92.84)	1782(93.84)	
Yes	157(6.39)	40(7.16)	117(5.16)	
Digestive system diseases				0.445
No	2215(90.11)	499(89.27)	1716(90.36)	
Yes	243(9.89)	60(10.73)	183(9.64)	
Dental/oral diseases				
No	2195(89.30)	479(85.69)	1716(90.36)	
Yes	263(10.70)	80(14.31)	183(9.64)	0.002
Denture use				< 0.001
chew with natural teeth	1555(63.26)	267(47.76)	1288(67.83)	
chew with false teeth	854(34.74)	257(45.97)	597(31.44)	
without natural and false teeth	49(2.00)	35(6.26)	14(0.74)	
Joint diseases				0.211
No	1980(80.55)	440(78.71)	1540(81.10)	
Yes	478(19.45)	119(21.29)	359(18.90)	
Fractures/osteoporosis				< 0.001
No	2019(82.14)	431(77.10)	1588(83.62)	
Yes	439(17.86)	128(22.90)	311(16.38)	
Kidney disease				0.007
No	2392(97.31)	535(95.71)	1857(97.79)	
Yes	66(2.69)	24(4.29)	42(2.11)	
Prostate disease				0.094
No	1051(94.68)	239(92.64)	812(95.31)	
Yes	59(5.32)	19(7.36)	40(4.69)	
Gynecological diseases				0.055
No	1271(94.29)	277(92.03)	994(94.94)	
Yes	77(5.71)	24(7.97)	53(5.06)	
Others				0.422
No	2290(93.17)	525(93.92)	1765(92.94)	
Yes	168(6.83)	34(6.08)	134(7.06)	
Number of diseases				0.000
0	362(14.73)	69(12.34)	293(15.43)	
1 ~ 2	1402(57.04)	287(51.34)	1115(58.72)	
3 ~ 5	591(24.04)	160(28.62)	431(22.70)	
> 5	103(4.19)	43(7.69)	60(3.16)	

Note: Hyperlipidemia: hypertriglyceridemia and hypercholesterolemia; Thyroid disease: hyperthyroidism and hypothyroidism; Mental illnesses: depression and schizophrenia; Eye diseases: glaucoma, cataracts, and retinopathy; Cardiovascular diseases: hypertension, angina pectoris and myocardial infarction; Respiratory system diseases: asthma and chronic obstructive pulmonary disease; Digestive system diseases: chronic gastritis, peptic ulcer, esophagitis, enteritis, cirrhosis, pancreatitis, and cholecystitis; Dental/oral diseases: periodontitis, denture use, periapical periodontitis, residual roots, and dentition loss; Joint diseases: rheumatoid joints and arthritis; Kidney disease: chronic urinary tract infections, chronic nephritis and uremia; Prostate disease: benign prostatic hyperplasia; Gynecological diseases: chronic cervicitis, vaginitis, pelvic inflammatory endometriosis, uterine fibroids, and dysfunctional uterine bleeding; Other diseases: anemia, malignant tumor, or trauma of other parts

To explore the association between medical history and the onset dementia, in Model 1, we controlled age, education, house work, social activity, spouse, smoking and drinking, it turned out that diabetes, dental/oral diseases and denture use were associated with dementia. Model 2 to Model 4 presented the effect of medical history on dementia in different age groups (Age: 60–70, 71–80, and > 81) after controlling for education, house work, social activity, spouse, smoking and drinking. In older adults aged 60–70, diabetes, mental illness, dental/oral diseases and denture use contributed to dementia. In older adults aged 71–80, only denture use was statistically significant to dementia. In model 4 (Age: > 81), dental/oral diseases and denture use were related to dementia (Table 3).

**Table 3:** Multivariate regression model for medical history to dementia after controlling variables (*n* = 2458)

	Model 1		Model 2		Model 3		Model 4	
	Overall		Age: 60–70		Age: 71–80		Age: > 81	
	*β*	*P*	*β*	*P*	*β*	*P*	*β*	*P*
Diabetes	0.248	0.048	0.510	0.043	0.348	0.084	-0.047	0.824
Thyroid disease	0.442	0.313	0.356	0.778	0.773	0.221	0.427	0.553
Mental illnesses	0.798	0.067	1.730	0.014	0.550	0.487	-0.270	0.751
Hearing loss	0.181	0.595	0.408	0.389	0.547	0.089	-0.068	0.827
Stroke	0.106	0.538	0.480	0.267	0.281	0.343	-0.069	0.770
Dental/oral diseases	0.311	0.047	0.814	< 0.001	-0.066	0.803	0.822	0.002
Denture use	0.473	< 0.001	0.701	0.003	0.428	0.006	0.531	< 0.001
Fractures/osteoporosis	0.231	0.069	0.320	0.247	0.171	0.427	0.113	0.578
Kidney disease	0.392	0.168	0.887	0.220	0.207	0.660	0.431	0.327
Control variables	yes		yes		yes		yes	

Note: Control variables include age, education, house work, social activity, spouse, smoking and drinking in Model 1. Model 2–4 present the effect of medical history on dementia in different age groups (Age: 60–70, 71–80, and > 81) after controlling for education, house work, social activity, spouse, smoking and drinking. Because of the small sample size in population more than 90 years old, we combined the age group of 81–90 and > 90

## Discussion

We explored the medical history systematically and determined the effect of medical history on the onset of dementia on the basis of community population sampling. Among all the diseases investigated, diabetes, thyroid disease, mental illness, hearing loss, stroke, dental/oral disease, denture use, fracture/osteoporosis, kidney disease, and number of diseases were risk factors for dementia. After controlling for the demographic sociological information, variables of diabetes, dental/oral disease and denture use were the main risk factors for dementia.

By multivariate regression, our study revealed that diabetes, dental/oral disease and denture use were independent risk factors for dementia. As for diabetes, it was morphologically related to neuronal loss in the frontal and temporal lobes, clinically, diabetic patients naturally showed decreased cognitive functions [39]. It has been revealed that cardiovascular risk factors, changes in insulin metabolism, glucose toxicity and inflammation may be associated with dementia [40]. What’s more, diabetes modified metabolism of Aβ and tau, leading to Aβ/tau-dependent pathological changes and dementia. Regarding dental/oral disease, previous literature [30] showed that the number of lost teeth was related to the incidence of dementia. Cognitive impairment was attributed to lack of oral hygiene, bacterial dental plaque accumulation and gingival inflammation [29]. Neuroinflammation activated the microglia and proinflammatory cytokines, and then triggered irreversible neurodegenerative deterioration [31]. Evidence implied the causal relationship between mastication and cognition in aging and dementia [28]. As to denture use, the concept of the ‘brain-stomatognathic axis’ assumes that the cognitive conditions contribute to oral sensorimotor function. On the one hand, a top-down control from the brain to the stomatognathic system has been established. A causal connection between a decrease in cognition and a chewing dysfunction has been demonstrated in animal research. On the other hand, it can be assumed that input from the stomatognathic system influences the brain. Denture use is due to clinical and epidemiological risk factors, discovered through animal studies, for the decline in cognitive functions [41, 42]. In subgroup analysis based on age, we found that dental/oral diseases and denture use contributed to dementia in nearly all age groups.

Multi-morbidity is a common situation among older adults. The older adults are more likely to be suffering from one or more co-existing diseases such as hypertension [43], diabetes [44], stroke [45], and respiratory system diseases [46], and these diseases have been proved to be related to dementia. In this present survey, people with cardiovascular disease accounted for 51.38%, diabetes patients accounted for 18.43%, and diagnosed fractures or osteoporosis accounted for 17.86%. 57% of the enrolled older adults were complicated with one or two kinds of diseases, and 24.04% of them suffered from three to five types of diseases. Our results suggested that the prevalence of dementia in the older adults with one to two diseases, three to five diseases and more than 5 diseases were 20.47%, 27.07% and 41.75% respectively. Most previous researches focused only on the impact of one specific disease on dementia [47–49]. However, according to our study, multimorbidity state was influential on dementia. Both cardiovascular disease and diabetes were considered as the upstream of observed cognitive decline [50, 51]. Dementia and these common chronic diseases among the older adults shared overlapping risk factors, comorbidities and pathophysiological mechanisms [52]. Moreover, neurodegeneration impaired the microcirculation, as a result of which the neurodegeneration accelerated [49]. Therefore, coexistence of multiple diseases produced a synergistic effect, accelerating the progress of dementia.

Though the effect of mental illnesses (*P* = 0.067) and fractures/osteoporosis (*P* = 0.069) on dementia did not reach the statistical significance in our final model, more attention should be paid to them. Osteoporosis was a common systemic skeletal disease that mainly affected older adults and co-occurred with dementia [53]. It led to bone fragility and susceptibility to fracture. Patients with osteoporosis were at 1.46-fold higher risk of dementia, and those who received bisphosphonate treatment or estrogen supplementation were at lower risk [54]. Risk factors of osteoporosis such as vitamin D, K and Calcium deficiency were all related to cognitive decline in the older adults. The most compelling evidence for the common etiology between osteoporosis and dementia lay in the APOE4 allele and amyloid beta disorders [55]. Some studies revealed that risks of dementia diagnosis are significantly higher for older adults diagnosed with schizophrenia, bipolar disorder and major depressive disorder [56, 57]. Therefore, although no positive results were found in our study, the association of mental illnesses and osteoporosis with dementia still warrants further attention. Further studies are needed to explore the relationship between thyroid disease, mental illnesses, hearing loss, stroke, fractures/osteoporosis, kidney disease and dementia [58].

### Implication to clinical practice

These findings implicated that having a good command of medical history made it possible to come up with novel treatment for dementia, by which medical staff may optimize their care for older adults who are at the risk of dementia. Previous researchers have demonstrated the correlation between dementia and one specific disease, our research systematically explored the effect of diseases on the risk of dementia, and determined the importance of the number of diseases. Our findings should serve as an incentive for government to modify the health practices and policies appropriately.

Management of hypertension and diabetes has been covered by the health care system in China. However, osteoporosis/fracture, dental/oral diseases have not been paid enough attention to till now. It is strongly recommended that the government health care system establish universal hospital insurance and management on the diseases prior to the onset of dementia. Although there is no cure for dementia, future research should focus on the overall health status of older patients with multiple coexistence diseases and the management on the changeable risk factors to delay the onset of dementia.

### Strength and limitation

The strengths of our research lay in the community population sampling and the focus on the coexistence of multiple diseases. A major limitation of this study was the evaluation of diseases history after the age of 40. The data was based on community record and the older adults’s self-report. It might cause recall bias to a large degree. In China, the government medical system has established a comprehensive screening and diagnostic system for cardiovascular diseases such as hypertension, diabetes, and stroke. Therefore, data of such diseases is accurate. However, other common disorders among older adults such as hearing loss, tooth loss, periodontitis and osteoporosis, are lack of sufficient public attention. A substantial proportion of older adults are still unaware of their impairment. Therefore, the incidence of these diseases in our study might be relatively lower than the actual status. This could lead to an underestimate of these diseases in the current study. Additionally, the small preclinical symptoms may have occurred long before the onset of dementia. Therefore, our cross-sectional design could not distinguish the causal relationship between diseases and dementia clearly. What’s more, in our study, dementia was evaluated by HDS-R without clinical diagnosis, which also had an effect on the result.

## Data Availability

All data generated or analyzed during this study are included in this published article.
